# A major quantitative trait locus affecting resistance to Tilapia lake virus in farmed Nile tilapia (*Oreochromis niloticus*)

**DOI:** 10.1038/s41437-021-00447-4

**Published:** 2021-07-14

**Authors:** Agustin Barría, Trọng Quốc Trịnh, Mahirah Mahmuddin, Carolina Peñaloza, Athina Papadopoulou, Ophelie Gervais, V. Mohan Chadag, John A. H. Benzie, Ross D. Houston

**Affiliations:** 1 The Roslin Institute and Royal (Dick) School of Veterinary Studies, University of Edinburgh Easter Bush, Midlothian, United Kingdom; 2 WorldFish, Bayan Lepas, Malaysia

**Keywords:** Quantitative trait loci, Quantitative trait, Genetic markers, Animal breeding, Genome-wide association studies

## Abstract

Enhancing host resistance to infectious disease has received increasing attention in recent years as a major goal of farm animal breeding programs. Combining field data with genomic tools can provide opportunities to understand the genetic architecture of disease resistance, leading to new opportunities for disease control. In the current study, a genome-wide association study was performed to assess resistance to the Tilapia lake virus (TiLV), one of the biggest threats affecting Nile tilapia (*Oreochromis niloticus*); a key aquaculture species globally. A pond outbreak of TiLV in a pedigreed population of the GIFT strain was observed, with 950 fish classified as either survivor or mortality, and genotyped using a 65 K SNP array. A significant QTL of large effect was identified on chromosome *Oni22*. The average mortality rate of tilapia homozygous for the resistance allele at the most significant SNP (*P* value = 4.51E−10) was 11%, compared to 43% for tilapia homozygous for the susceptibility allele. Several candidate genes related to host response to viral infection were identified within this QTL, including *lgals17*, *vps52*, and *trim29*. These results provide a rare example of a major QTL affecting a trait of major importance to a farmed animal. Genetic markers from the QTL region have potential in marker-assisted selection to improve host resistance, providing a genetic solution to an infectious disease where few other control or mitigation options currently exist.

## Introduction

Aquaculture is the fastest-growing food production sector worldwide, with an average growth in production of 5.3% per annum during the period 2001–2018 (FAO 2020). Nile tilapia (*Oreochromis niloticus*) has the third-highest production of all finfish species, with more than 4.5 million tons farmed in 2018 (FAO 2020). Tilapia aquaculture represents a critical source of protein and nutrients for human consumption in many low and middle-income countries across Africa, Asia, and America. However, the recent emergence of Tilapia lake virus (TiLV) has presented major threats to the sustainability of tilapia production, affecting both Nile tilapia and interspecific hybrids (Eyngor et al. 2014; Ferguson et al. 2014; Dong et al. 2017), with mass mortalities up to 90% possible at several stages of the production cycle (Dong et al. 2017; Fathi et al. 2017). This disease is produced by an RNA virus which has recently been classified as a member of the *Tilapinevirus* genus, *Amnoonviridae* family (ICTV 2018). There is evidence that TiLV can be transmitted both vertically and horizontally (Eyngor et al. 2014; Jaemwimol et al. 2018; Dong et al. 2020), highlighting the importance of TiLV-free broodstock. Furthermore, there are currently only a few treatments and options for prevention, and with outbreaks possible in very young juvenile fish, the potential of vaccination is limited (Thammatorn et al. 2019).

Improvement of host resistance to infectious disease in farmed fish species via selective breeding has major potential to prevent outbreaks and improve the sustainability of production, with some notable success stories to date (Yañez et al. 2014; Gjedrem 2015; Houston 2017). Encouragingly, a recent study by our team detected significant and high heritability for host resistance to TiLV in tilapia from a commercial breeding program, as measured by survival during a pond outbreak (Barría et al. 2020). However, while pedigree-based breeding programs are effective at improving target traits, the use of genomic tools can expedite sustainable genetic gain in aquaculture (Houston et al. 2020). The availability of genomic tools for a variety of aquaculture species (e.g. Houston et al. 2014; Palti Gao et al. 2015; Lien et al. 2016; Yáñez et al. 2016; Gutierrez et al. 2017; Zeng et al. 2017; Nugent et al. 2019; Yu et al. 2020; Zhou et al. 2020) has also facilitated the identification of quantitative trait loci (QTL) associated with traits such a sexual determination, growth rate, flesh color and disease resistance which are common targets for commercial breeding programs (e.g. Gonen et al. 2015; Palti Vallejo et al. 2015; Tsai et al., 2015; Gutierrez et al. 2018; Mohamed et al. 2019; Fraslin et al. 2020).

The most remarkable example is the identification of a major QTL affecting host resistance to infectious pancreatic necrosis virus (IPNV) in two different Atlantic salmon (*Salmo salar*) populations, explaining between 80 and 100% of the genetic variation for the trait (Houston et al. 2008,2010; Moen et al. 2009). Application of favorable alleles through marker-assisted selection (MAS) has resulted in the number of IPN outbreaks in Norway reducing to near zero (Hjeltnes 2014; Norris 2017). However, QTL of such large effect are rare, and for traits underpinned by a polygenic genetic architecture, genomic selection (GS) is a more effective approach (Zenger et al. 2019; Houston et al. 2020).

The development and application of genomic tools have been relatively recent for Nile tilapia. However, there is a high-quality reference genome (Conte et al. 2019) and at least three different medium to high-density SNP arrays (Joshi et al. 2018; Peñaloza et al. 2020; Yáñez et al. 2020). These resources have already been used to highlight the potential improvement in accuracy of prediction of estimated breeding values (EBVs) for growth and disease-resistance related traits compared with a pedigree-based approach (Yoshida Lhorente et al. 2019; Joshi et al. 2020; Joshi Skaaurd 2020), and have also allowed the assessment of linkage disequilibrium (LD) and genetic structure of different GIFT-derived Nile tilapia populations (Yoshida et al. 2019).

Due to the global importance of TiLV, and the evidence for significant and high levels of heritability for host resistance (Barría et al. 2020), there is a major opportunity to utilize genomic tools to investigate the genetic architecture of this resistance. As such, the aims of this study were to (i) dissect the genetic architecture of resistance to TiLV using a high-density SNP array, (ii) map QTLs associated with host resistance to regions of the tilapia genome, and (iii) identify positional candidate genes within those QTL regions that may be associated with this trait. The results of this study will inform the use of genetic markers in tilapia breeding programs aiming to develop tilapia strains with enhanced resistance to TiLV. Furthermore, the identification of candidate genes will lead to research targeting improved knowledge of the functional mechanisms underlying resistance to TiLV, hence offering the opportunity for the development of new control and treatment strategies.

## Material and methods

### Nile tilapia population

The population sample used in this study is from a genetically improved Nile Tilapia (GIFT) breeding program and had been selected for improved growth rate for 16 generations. The breeding nucleus was based in Jittra, Malaysia, and managed by WorldFish (Penang, Malaysia). The population sample consisted of 124 nuclear families (GIFT generation 16th) produced by crossing 124 dams and 115 sires. Pedigree data for these fish included records of approximately 86,000 fish from generation 16 back to the first generation of GIFT in Malaysia. Each fish from the current generation was tagged using a passive integrated transponder (PIT tag) at an average weight of 4.97 g, which corresponded to an average age of 110.5 days post-hatching. At typical harvest weight, fish were transferred to a single pond where a natural TiLV outbreak was observed shortly thereafter. A summary of the phenotypes measured on these fish, such as weight and age at tagging and harvest for this population can be found in Barría et al. (2020).

### Natural field outbreak

After the fish were transferred to the single pond, a natural field outbreak of TiLV was observed (in February and March 2018). Mortalities were collected and sampled daily for 14 days (22 February to 7 March 2018), and once the mortality levels had returned to baseline, all remaining fish in the pond were euthanized (using 400 mg/l clove oil) and sampled over five days (8–12 March 2018). A total of 1,821 fish were classified as survivors or mortalities, and the phenotypic sex of all fish was determined. On average, each full-sibling family comprised 14 fish (ranging from 2 to 21). Clinical signs of TiLV were observed throughout the outbreak. The presence of TiLV was assessed on the spleen of a subset of 35 fish, through one-step RT-qPCR. Briefly, the EvaGreen assay RT-qPCR was performed using the following primer set; Forward primer: 5′–CTGAGCTAAAGAGGCAATATGGATT–3′ and Reverse primer: 5′–CGTGCGTACTCGTTCAGTATAAGTTCT–3′. Finally, the PCR product was detected by measuring the fluorescence generated by the EvaGreen dye bound to dsDNA. More details about this RT-qPCR assay, including reproducibility and sensitivity, can be found on Tattiyapong et al. (2018). A sample of mortalities was also randomly selected to perform necropsy analyses to further confirm TiLV as the cause of the mortalities. In addition, a caudal fin sample was taken from all survivors and mortalities from the outbreak, placed in 95% ethanol, and stored at −20 °C until further DNA extraction and genetic analysis.

### Genotyping

Total DNA from fin clips of 2,016 fish, including 195 parents and all the 1,821 offspring collected from the outbreak, was extracted using a salt-extraction protocol (Aljanabi and Martinez 1997), with the modifications described by Taslima et al. (2016). The DNA quality and/or quantity from 691 offspring was not sufficient to surpass the initial quality control (QC) criteria required by the genotyping company (Identigen, Dublin, Ireland). Therefore, only 1,130 offspring and the 195 parents were genotyped. The extracted DNA samples were genotyped using an Axiom® SNP array developed by our team which contains ~65 K SNP markers dispersed throughout the Nile tilapia genome (Peñaloza et al. 2020). The raw array data from the genotyping (CEL intensity files) were imported to the Axiom analysis Suite v4.0.3.3 software for genotype calling and QC. A total of 47 samples with a dish quality control (DQC) and call rate (CR) <0.82 and <0.93, respectively, were excluded for subsequent analyses. Thus, 187 parents (96%) and 1,091 offspring (97%) passed the thresholds of the Axiom software. Approximately 54 K (78%) of the SNPs on the array were identified as high quality and polymorphic (i.e., “PolyHighResolution”) and were retained for further analyses (Peñaloza et al. 2020). Subsequently, a second QC step was applied using Plink v1.09 (Purcell et al. 2007). With an average CR of 99%, all fish surpassed the genotype call rate (>0.95). The SNPs with a minor allele frequency (MAF) < 0.05, CR < 0.95, and significant deviation from Hardy–Weinberg equilibrium (HWE) (*p* < 1 × 10^−6^) were excluded from further analyses. Thus, 94% of the SNPs (50,710 out 53,811) passed all the QC filters, with most of them being removed due to low MAF (~2 K SNPs). Furthermore, using trio information, the resulting data set was tested for putative Mendelian errors in any fish and SNPs. A total of 166 fish (25 parents and 141 offspring) and 3 K SNPs were excluded for subsequent analyses due to a Mendelian error rate >5%, which could reflect either errors in pedigree recording or samples with poor quality genotype data. Following all QC steps, the final filtered dataset comprised 1,112 fish and 47,915 SNPs. This comprised genotype and phenotype data for 950 offspring, and genotype data for 162 parents. From this filtered dataset, the genotyped offspring represent fish from 117 families (108 full-sib families), with an average of 8 fish per family, ranging from 1 to 16.

### Estimation of genetic parameters

Host resistance to TiLV was defined as binary survival (BS) (i.e., dead/alive at the end of the natural field outbreak) and as a time to death (TD). In the case of BS, the survivors and mortalities were designated as 1 or 0, respectively. The TD trait was considered as a continuous trait, with values ranging from 1 up to 14, representing the day of the first observed mortality and the last day before mortality returned to baseline levels, respectively. The heritability for BS and TD in the genotyped population was estimated using a genomic-relationship matrix (GRM) calculated with the genome-wide complex trait analysis (GCTA) software v.1.92.2 (Yang et al. 2011). All SNPs surpassing the QC were used to create the GRM. This matrix was then used to estimate the narrow-sense heritability by using the following linear model:

1 $$y = \mu + {{X}}b + {{Z}}u + e$$

Where *y* is the vector of phenotypes (BS or TD records), *μ* is the population mean, *b* is the vector of fixed effects (sex as fixed effect, and weight and age at harvest as covariates), *u* is the vector of the additive genetic effects, and *X* and *Z* are incidences matrices. The following distributions were assumed; ${{u}}\sim {{N}}\left( {0,{{G}}\sigma _{{u}}^2} \right)$ and ${{e}}\sim {{N}}\left( {0,{{I}}\sigma _{{e}}^2} \right)$. Where $\sigma _{\boldsymbol{u}}^2$ and $\sigma _{\boldsymbol{e}}^2$ are the additive genetic and residual variance, respectively, *G* is the GRM and *I* is the identity matrix. Heritability was estimated as the ratio of the additive genetic variance to the phenotypic variance. A genetic correlation was estimated as the ratio of the genetic covariance between BS and TD to the square root of the product of the genetic variance of BS and TD.

### Genome-wide association study (GWAS)

To identify SNPs associated with TiLV resistance (defined as both BS and TD), a mixed linear model using the *leaving-one-chromosome-out* (LOCO) approach was applied using the GCTA v.1.92.2 software. This approach estimates the GRM between individuals by removing the SNPs located in the tested chromosome and including SNPs from all the other chromosomes. The SNP effect from the chromosome being tested is not included twice in the model, and therefore a higher statistical power is achieved (Van den Berg et al. 2019). Subsequently, fitting the GRM allows correction for population structure, which can cause spurious associations in GWAS. The model used for the GWAS was identical to the model described previously for the estimation of the genetic parameters. However, the specific SNP genotypes being assessed were coded as 0, 1, or 2 (reflecting the number of minor alleles at the locus), and included in the model as random effects (Yang et al. 2011). For an SNP to be considered significant at the genome-wide level, it had to surpass the genome-wide Bonferroni-corrected significance threshold for multiple testing, which was defined as *P* < 0.05/47,915. This multiple test correction is considered very stringent (Johnson et al. 2010), which reduces the likelihood of any false positive association. To quantify the level of inflation of the obtained *P*-values compared with those expected, lambda (λ) was computed as the median of the quantile χ^2^ distribution of the obtained *P*-values/0.455. For practical reasons, SNPs not placed in chromosomes in the reference genome assembly (O_niloticus_UMD_NMBU, Genbank accession number GCA_001858045.3, Conte et al. 2019), were assigned as being placed on a “psuedo-chromosome” *Oni24*. GWAS results were plotted using the package “CMplot” (Yin 2020) in R (R Core Team 2017).

### Candidate genes in QTL regions

Based on the genome-wide association results, putative candidate genes associated with host resistance to TiLV were identified within a 1 Mb window size (500 Kb upstream and downstream) flanking the significantly associated SNPs, again using the Nile tilapia reference genome assembly (Genbank accession number GCA_001858045.3).

### Estimation of SNP variances

Following the GWAS, the top three SNPs significantly associated with BS and/or TD on each of the significant chromosomes were tested for the estimation of the additive and dominance effect, by using ASReml v.4.1.0 (Gilmour et al. 2015). Additive (*a*) and dominance (*d*) effect were estimated as follows: *a* = (AA – BB)/2 and *d* = AB – [AA + BB/2] where AA, AB, and BB are the predicted trait value for each genotype. The proportion of genetic variance explained for each of the selected SNPs was estimated as [2*pq*(*a* + *d*(*q* – *p*))2]/VA, where p and q are the frequencies of the SNP alleles, and VA is the total additive genetic variance explained by the model when no individual SNP is fitted.

## Results

### Field outbreak

Throughout the TiLV outbreak, clinical signs related to this viral infection were observed, including damage at the base of fins, hemorrhage, and skin erosion. These were confirmed by a qualified veterinarian. Subsequently, through an RT-qPCR assay, TiLV was identified in 74% of the randomly sampled fish (26 out of 35). From the 35 sampled fish, 15 were survivors and 20 were mortalities, with 40% of the survivors testing positive and 100% of the mortalities testing positive. The survivors which tested positive had a substantially lower viral load, with 127 viral copies/μl ± 150, compared to 188,491 ± 233,167 copies/μl for mortalities.

After 14 days of the first observed mortality, the daily mortality level had returned close to the baseline, and all remaining fish in the pond were euthanized and sampled. The total cumulative mortality in the outbreak in the entire sampled population was 39.6%. Further details about the pattern of mortality during the TiLV outbreak can be found in Barría et al. (2020).

### Genome-wide association study

The estimates of heritability in this dataset based on the GRM, were consistent with the estimates using the pedigree on a larger dataset described in Barría et al. (2020). Moderate to high heritability values of 0.38 ± 0.05 and 0.69 ± 0.09 were estimated for BS on the observed and underlying scale, respectively, whereas a lower value was estimated for TD (0.22 ± 0.05). The robustness of the estimation of the variance parameters for BS on the underlying scale was confirmed by implementing a threshold model in the Thrgibbs1f90b module of the Blupf90 (Misztal et al. 2015) software (data not shown).

**Fig. 1:**
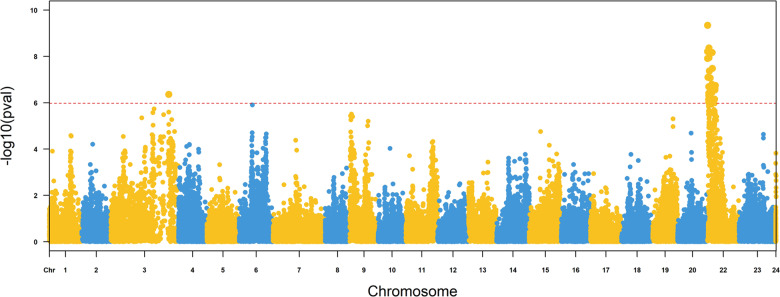
Manhattan plot for resistance to Tilapia lake virus (TiLV) in a Nile tilapia (*Oreochromis niloticus*) breeding population. Manhattan plot of GWAS for host resistance, as binary survival, to TiLV. On the *y* axis is the –log10(*P*-value). The horizontal dashed red line shows the genome-wide significance threshold. *Oni24* represent SNPs with unknown chromosome location.

**Fig. 2:**
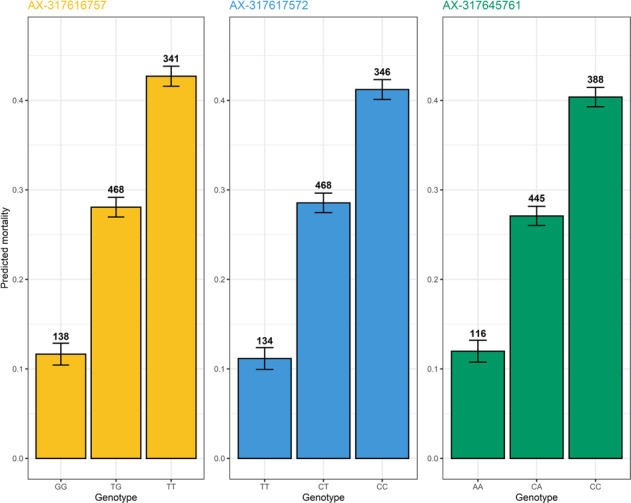
Predicted mortality values for host resistance to Tilapia lake virus in a Nile tilapia breeding population. Host resistance as binary survival predictive values for each genotype of the SNPs with the stronger genome-wide association. The bars on yellow, light blue, and green show the predicted values for the top three SNPs located on *Oni22*. The bars show the standard error. Numbers above the bars indicate the number of fish with the specific genotype.

A high genetic correlation was found between both TiLV resistance definitions (0.97 ± 0.02, Table 1). A total of 29 SNPs that exceeded the genome-wide significance Bonferroni threshold for BS were identified, and all but 1 were located on chromosome *Oni22* (Table 2), with a clear peak suggestive of a major QTL in the proximal end of this chromosome (Fig. 1). In the case of TD, two SNPs located in the same QTL region of *Oni22* surpassed this genome-wide significance threshold (Fig. S1). The estimated inflation factor (λ) for BS and TD was 1.19 and 1.11, respectively, suggesting a relatively good concordance between the observed *P*-values and the theoretical statistical distribution. The QQ plot associated with the Manhattan plot for both traits is shown in Fig. S2. Interestingly, for both definitions of resistance, the most significant association was found for the same SNP (AX-317616757, located at the position 255,104 bp on *Oni22*) with a *P*-value of 4.5 × 10^−10^ and 4.8 × 10^−07^, for BS and TD, respectively (Table 2). All the SNPs located in *Oni22* which were significantly associated with BS are within a genomic region of approximately 9.4 Mb in size. However, this QTL size is reduced to ~1.7 Mb when only the top three SNPs are taken into account (AX-317645761 and AX-317617572 located at 239,073 and 1,939,192 bp respectively). The minor allele was associated with resistance to TiLV for 25 out of the 29 significant SNPs. The frequency of these resistance-associated alleles ranges from 0.11 to 0.48 (Table 2), highlighting the potential for increasing its frequency by MAS and hence TiLV resistance at a population level. In the case of the three SNPs with the lowest P values, the proportion of genetic variance explained ranged from 0.06 to 0.14 (Table 3). The three most significant SNPs on *Oni22* have a substitution effect on TiLV mortality proportion ranging from 0.16 to 0.14 (Table 3 and Fig. 2). For example, at the most significant SNP, the average mortality rate of tilapia carrying two copies of the resistance allele was 11%, compared to 43% for tilapia carrying two copies of the susceptibility allele. Therefore, the predicted difference in mortality between alternate homozygous fish at this single significant QTL is 32%, which can be placed in context by considering that the overall mortality rate in the outbreak was ~40%. In the case of the significant SNP located on *Oni03* (AX-317718855), the equivalent allele substitution effect is 0.07.

**Table 1:** Genetic parameters for host resistance to TiLV in a Nile tilapia (*Oreochromis niloticus*) breeding population.

Parameters^b^	BS^a^	TD^a^
$\sigma _a^2$	0.09(0.02)	5.96(1.47)
$\sigma _e^2$	0.14(0.01)	21.6(1.33)
$\sigma _p^2$	0.22(0.01)	27.57(1.39)
*h*^2^	0.38(0.05)	0.22(0.05)
*r*_*g*_	0.97(0.02)	

Standard errors are shown inside brackets.

^a^Host resistance definition: *BS* binary survival on the observed scale, *TD* time to death.

^b^Genetic parameters and standard error: $\sigma _a^2$ = additive genetic variance; $\sigma _e^2$ = error variance; $\sigma _p^2$ = phenotypic variance; *h*^2^ = narrow-sense estimated heritability; *r*_*g*_ = genetic correlation.

**Table 2:** Significant SNPs associated with TiLV resistance as binary survival (BS) and time to death (TD) in a Nile tilapia (*Oreochromis niloticus*) breeding population.

SNP	*Oni*^a^	BP^b^	*P*-value^c^	Minor/major allele^d^	Resistance allele freq^f^
***TD***					
AX-317616757	22	255104	4.80E−07	**G**/T	0.39
AX-317647630	22	5379664	8.20E−07	**G**/A	0.11
***BS***					
AX-317616757	22	255104	4.51E−10	**G**/T	0.39
AX-317617572	22	1939192	4.32E−09	**T**/C	0.39
AX-317645761	22	239073	6.17E−09	**A**/C	0.36
AX-317619074	22	5785936	6.86E−09	**G**/A	0.37
AX-317617531	22	1888302	1.13E−08	**G**/A	0.47
AX-317616778	22	276593	1.24E−08	A/**G**	0.56
AX-317648645	22	6115097	3.30E−08	**A**/G	0.46
AX-317617852	22	2432164	4.23E−08	**T**/G	0.40
AX-317616808	22	760642	7.96E−08	**T**/C	0.48
AX-317647368	22	3478052	8.47E−08	**A**/G	0.48
AX-317647975	22	5629013	1.60E−08	**G**/T	0.16
AX-317621349	22	9718148	1.81E−08	G/**A**	0.72
AX-317617390	22	1758060	2.02E−08	**C**/T	0.42
AX-317647016	22	2521284	2.13E−08	**A**/G	0.14
AX-317618485	22	5289967	2.22E−08	**T**/C	0.39
AX-317649969	22	9299308	2.38E−08	A/**G**	0.58
AX-317647630	22	5379664	2.70E−08	**G**/A	0.11
AX-317620863	22	9336674	2.81E−08	**C**/T	0.45
AX-317647753	22	5479097	3.56E−08	**C**/A	0.11
AX-317617254	22	1616487	3.92E−08	**C**/T	0.11
AX-317718855	03	71847333	4.37E−07	C/T^e^	0.24
AX-317646938	22	2445275	4.45E−07	**A**/G	0.10
AX-317646411	22	1714252	4.95E−07	**A**/G	0.10
AX-317617977	22	2555673	4.97E−07	**C**/T	0.14
AX-317620317	22	8916395	6.99E−07	**C**/T	0.13
AX-317063470	22	1441242	7.27E−07	**A**/G	0.10
AX-317646673	22	1988041	7.86E−07	**A**/G	0.29
AX-317617288	22	1660924	9.54E−07	**T**/G	0.11
AX-317648270	22	5898003	9.65E−07	**G**/A	0.12

^a^Number of chromosome on the *Oreochromis niloticus* genome.

^b^Position of the SNP in the chromosome, in base pairs.

^c^*P* value of the SNP for the genome-wide association study for host resistance to TiLV.

^d^In bold is the allele conferring the resistant phenotype.

^e^The resistant genotype is heterozygous.

^f^Frequency of the allele conferring the resistance phenotype to TiLV.

**Table 3:** Summary statistics for the most significant genome-wide associated SNPs within each chromosome for host resistance to TiLV.

*Oni*^a^	SNP	BP^b^	*a*^c^	*P* value (a)^d^	Vg
***Binary survival (BS)***					
22	AX-317616757	0.25	0.16	2.45E−09	0.12
22	AX-317617572	1.93	0.15	1.19E−08	0.10
22	AX-317645761	0.23	0.14	1.90E−07	0.09
3	AX-317718855	71.8	0.07	8.42E−02	0.06
***Time to death (TD)***					
22	AX-317616757	0.25	−1.37	3.12E−06	0.14
22	AX-317647630	5.37	−1.97	5.45E−02	0.13

^a^Number of chromosome on the *Oreochromis niloticus* genome.

^b^Position of the SNP in the chromosome, in million base pairs.

^c^Additive genetic effect.

^d^*P* value of the Student’s *t*-test distribution for the genetic effect.

^e^Proportion of the genetic variance explained by the SNP.

### Candidate genes in the QTL regions

The SNP with the lowest *P* value for BS and TD is located within the second intron of the *lgals17* gene, a member of the galectin family. This family of genes has functions that include antiviral defense. A number of other interesting candidate genes were also located within this QTL region, which has previously been found to be related to host response to a viral infection. For the main QTL on *Oni22* the genes *rnf2, vps52*, *cdc42*, and *anp32* were identified. For the secondary QTL on *Oni3*, the *zbed1* (also known as *dref)*, *trappc1*, and *psmb6* were identified. The other SNP found to be associated with TD and BS (AX-317647630) is flanked by two genes belonging to the tripartite motif family, *trim21* and *trim29*.

The complete list of genes flanking the SNPs with the strongest association, within each chromosome, for host resistance to TiLV and their position within the QTL regions, are shown in Table 4.

**Table 4:** Genes flanking the most important genome-wide associated SNPs within each chromosome for TiLV resistance.

*Oni*^a^	Trait	QTL region^b^		Gene names
		Left position	Right position	
03	BS	71,347,333	72,347,333	**zbed1**^**c**^, kcnab1, trappc1, nlrc3, **psmb6**, pigr, **nlrc3**, **mrc1**, ephb4, **fcgr2b**, agp4, **btnl2**, **btnl10**
22	BS and TD	1	755,104	zhx1, **lgals17**, **vps52**, hmcn1, senp1, **muc5ac**, ha1f, **rnf2**, atad2, rps18, ptk7
22	BS	1,439,192	2,439,192	**zbed1**, zscan2, tmem65, sema6d, scgn, tatdn1, pomc, NADH, mtss1, grik5, fibcd1, carmil1, rnf139, ceacam5, **atx2**
22	BS	1	739,073	zhx1, **igals17**, **vps52**, hmcn1, senp1, **muc5ac**, ha1f, **rnf2**, atad2, rps18, ptk7
22	BS and TD	4,879,664	5,879,664	cacgn7, cacgn6, cacgn4, vamp2, styk1, **trim29**, tpi1, **glut1**, phc1, iffo2, **gnb3**, gapdh, gtan, eno2, g2e3, **trim21**, cox6b1, chd4, clcn1, **cdc42**, cd209e, **clec6a**

^a^Number of chromosome on the *Oreochromis niloticus* genome.

^b^QTL region size was defined as 500 kb upstream and downstream from the SNP location.

^c^In bold the name of the genes with a role known to be involved in a viral infection process.

## Discussion

### Data collection and genomic information

Experimental challenges are commonly used as a means to collect measurements of host disease resistance for the purposes of genetic improvement in aquaculture (Ødegård et al. 2011; Yañez et al. 2014; Houston et al. 2020). This is currently difficult for TiLV because experimental challenge models are at a formative stage and not yet routine in breeding programs. However, data and samples from field outbreaks can also be applied for the same purpose if opportunistic sampling can be performed during the course of the outbreak. The data and samples derived from such outbreaks have previously been used to estimate genetic parameters for disease resistance (Lillehammer et al. 2013; Bangera et al. 2014). In addition, when combined with genomic data, the resulting data can highlight QTL linked to host resistance in a field outbreak (Houston et al. 2008; Boison et al. 2019; Aslam et al. 2020), which is potentially very informative since the trait is a closer representation of the ultimate target trait in the breeding program, whereas equivalent results from experimental challenges would ideally need to be validated in a field setting.

### Heritability and genetic correlation

Together with our recent study (Barría et al. 2020), the current results provide substantial evidence supporting the feasibility to improve host resistance to TiLV by means of selective breeding, and the potential of genomic tools to assist and expedite this process. Previous heritability estimates for TiLV resistance, using pedigree relationship matrices, ranged from 0.40 to 0.63 for BS depending on the statistical model used. By using genomic relationship matrices, moderate to high heritabilities were estimated for BS within the same population (0.38 and 0.69 for the observed and underlying scale, respectively). These results highlight that rapid genetic gain should be possible by selection for host resistance, using either pedigree or genomic data to model relationships between animals.

A large number of studies have estimated significant heritabilities for host disease resistance in aquaculture species (reviewed in Yañez et al. (2014) and Ødegård et al. (2011)). In the case of Nile tilapia, and by using different statistical models, these estimates range from 0.11 to 0.58 and from 0.14 to 0.30 for the bacterial pathogens Streptoccocus sp (LaFrentz et al. 2016; Shoemaker et al. 2017; Suebsong et al. 2019; Sukhavachana et al. 2019; Joshi Skaaurd et al. 2020), and Flavobacterium columnare (Wonmongkol et al. 2017), respectively. Thus, our results are within the ranges of previous findings relating to disease resistance in farmed Nile tilapia populations. Furthermore, the magnitude of the genetic correlation between resistance defined as BS and resistance defined as days to death (0.97) is similar to the 0.95 estimated previously by using pedigree data (Barría et al. 2020). Similar high positive genetic correlations have been observed for host resistance traits defined as continuous or binary traits (Barría et al. 2018; Bassini et al. 2019), which is likely to be due to the fact that survivors at the end of the experimental challenge or natural field outbreak are typically assigned the same high value as for the days to death trait.

By using pedigree data, a genetic correlation not different from zero was estimated between harvest weight and TiLV resistance (Barría et al. 2020). The use of genomic data corroborated these results (0.06 ± 0.12 and 0.13 ± 0.14 for BS and TD regarding HW, respectively). Likewise, there was no evidence for the association of the TiLV-resistance-associated SNPs with harvest weight. Therefore, increasing the frequency of the resistant alleles within the population via family selection or MAS, should not affect genetic gain for body weight at harvest.

### Genome-wide association study

The GWAS comprised 950 fish and 48 K SNPs, which is comparable with previous studies in aquaculture species aimed to identify genomic regions associated with host resistance to infectious diseases (Gutierrez et al. 2018; Palaiokostas Cariou et al. 2018; Palaiokostas Robledo et al. 2018; Rodríguez et al. 2019; Vallejo et al. 2019; Fraslin et al. 2020). The genetic architecture of resistance to disease in aquaculture species varies substantially, from highly polygenic with no evidence for significant QTLs, to a single major QTL explaining almost all genetic variation in the trait (Fraslin et al. 2020). In the current study, the mapping of several highly significant SNPs to the proximal end of chromosome *Oni22* highlights an interesting QTL associated with resistance to TiLV and another QTL of potential interest on *Oni3*. For the main QTL, the size of the effect is highlighted by the >30% difference in mortality rate between tilapia of alternate homozygous genotypes, which points to the utility of QTL region SNPs in MAS. Therefore, the results suggest that resistance to TiLV in this population of GIFT tilapia is an oligogenic trait, with at least one likely major QTL together with minor effect QTL elsewhere in the genome. It is plausible that additional QTL would be detected with larger sample size and increased statistical power. Furthermore, relatively low levels of LD were detected in the parental population of the fish used for the GWAS (Peñaloza et al. 2020), making it difficult to exclude the possibility of additional QTL that were not in LD with SNPs included on the SNP array.

The presence of a major QTL underlying a quantitative trait is scarce in animal breeding populations. To date, most of the production traits of commercial interest in aquaculture have been found to be controlled by several loci with minor effects (Fraslin et al. 2020; Houston et al. 2020). However, there are some exceptions, and interestingly, with exception of age at maturity (Sinclair-Waters et al. 2020), these examples are primarily associated with host resistance to viral diseases in Atlantic salmon. For example, Gonen et al. (2015) found a major QTL, located on Atlantic salmon chromosome 3, for host resistance to salmonid alphavirus, using data collected from an experimental challenge. Boison et al. (2019) identified a major QTL for the piscine myocarditis virus located on Atlantic slmon chromosome 27 using data from a field outbreak. However, the most remarkable example is the discovery of a major QTL for resistance to IPN virus using data collected from a natural field outbreak (Houston et al. 2008) in post-smolts and then confirmed through an experimental challenge (Moen et al. 2009) in fry. One possible explanation for the existence of several major QTL affecting disease resistance in farmed fish populations is their recent domestication, such that these disease pressures are new, and standing genetic variation associated with resistance has not yet been subject to natural or domestication selection (Houston et al. 2020).

Given the large effect of the QTL mapped in the current study, it is likely that MAS for the favorable allele at the *Oni22* QTL will be of substantial utility for breeding tilapia with improved innate resistance to TiLV. However, due to variation in allele frequencies and genetic background, it would be beneficial to confirm these findings in other Nile tilapia breeding populations prior to implementation in specific populations. Ideally, an independent population should be used to evaluate if the same QTLs proposed here, or if new associations are detected. For example, previous reports suggest an impact of genetic background on resistance to TiLV, with the Chitralada strain being more susceptible than GMT and GIFT strains of Nile tilapia (Ferguson et al. 2014; Kabuusu et al. 2018). Another important future goal is to assess whether the QTL detected here is found under experimental conditions. If intraperitoneal injection is used as the challenge model, the innate immune response of the mucosal surfaces is largely bypassed, and therefore the host resistance trait may differ. This is important for facilitating future routine challenge testing of tilapia breeding populations for selective breeding purposes, and also for allowing further study of the underlying mechanisms of the QTL.

### Candidate genes

Due to the relatively recent discovery of TiLV, there is a relative paucity of information about the entry, dissemination, and replication of the virus, and the host immune response. In the current study, the SNP with the lowest *P* value for BS and TD (AX-317616757) is located at a position of 0.25 Mb on *Oni22*, specifically within the second intron of the *lgals17* gene. This gene encodes one of a family of proteins with functions as pattern-recognition receptors (PRRs) which are important in viral infections due to their role in activating the innate and adaptive immune response (Sato and Nieminen 2002; Vasta 2009). Galectins have shown increased expression and potential antiviral activity after exposure of several fish species to single-stranded RNA viruses, such as infectious salmon anemia virus (Jørgensen et al. 2008), viral hemorrhagic septicemia virus (VHSV) (*Haliotis discus*) (O’Farrell et al. 2002; Sandamalika and Lee 2020) and sea bass nervous necrosis virus (SBNNV) (Poisa-Beiro et al. 2009). Considering the homology of the largest genomic segment of TiLV with the PB1 subunit of the Influenza C virus polymerase (Bacharach et al. 2016), it is promising that *lgals1* has been found to reduce influenza virus load and that knockout mice have higher susceptibility than their wild-type counterparts (Yang et al. 2011).

Another gene of interest proximal to the SNP with the lowest *P* value is the *vps52 subunit of* the *GARP complex* gene. This gene is required for the replication and extracellular formation of two DNA viruses; vaccinia virus and monkeypox virus, and plays a crucial role in virus egress and cell-to-cell spread (Harrison et al. 2016; Realegeno et al. 2017). In addition, the *rnf2* gene is located within the QTL region and has been linked to inhibition of *ifn1*, which plays a crucial role in the innate response by inducing multiple antiviral genes in mice (Liu et al. 2018).

The *cdc42* gene is also located in the major QTL region, and several RNA viruses are known to hijack the actin-regulating pathways to internalize into human cells (Swaine and Dittmar 2015). Furthermore, the role of *cdc42* in the host response to viral infection has been observed in some aquaculture species. For example, Xu et al. (2017) and Levican et al. (2017) showed the ability of *cdc42* to inhibit the replication of the white spot syndrome virus and IPNV, respectively. Studies in Atlantic salmon populations revealed that this gene mapped to a QTL associated with host resistance to cardiomyopathy syndrome (Boison et al. 2019), and was also to be up-regulated in salmon with high levels of genetic resistance to amoebic gill disease (Robledo et al. 2020). The genes *trim29* and *trim21* were also located within the significant QTL. The former negatively regulates the IFN production in response to an infection mediated by RNA virus (Xing et al. 2018), while the latter is mainly associated with neutralization of the virus, operating during both the early and the late stages of the infection (Mallery et al. 2010; Vaysburd et al. 2013; Foss et al. 2019). Furthermore, it has been shown that *trim29*^−/−^ mice have higher survival and body weight than their wild-type counterparts following the influenza challenge (Xing et al. 2016).

A recent study performed by Wang et al. (2020), examined the host response to TiLV infection in an experimental challenge of Nile tilapia. They identified several genes and pathways upregulated after infection and identified enrichment of several pathways such as PPAR signaling, sucrose metabolism, phagosome, and cytokine-cytokine receptor interaction. None of the genes mapping to the identified QTLs in the current study were identified as being upregulated in response to infection in the study of Wang et al. These discrepancies could be due to a number of reasons, including the different nature of the infection. Whereas our data was obtained through a natural field outbreak, Wang et al. (2020) performed an IP experimental challenge. The latter could have bypassed the underlying factors related to the innate immune function of the mucosal layers and may have affected the host’s ability to mount a complete immune response to TiLV. Secondly, it is plausible that the causative gene underlying the QTL identified herein may not itself be differentially expressed, but may act via changes to the structure and function of key proteins involved in the host response. Further characterization of the genes and polymorphisms in the QTL region using whole-genome resequencing will help to identify potential underlying mutations for further study.

### Applications to tackle TiLV outbreaks

The presence of a large QTL underlying an important production trait is a relatively rare situation in farmed animal breeding. However, its usefulness has been effectively exploited in the aquaculture industry, by reducing the IPN-related mortalities in Norwegian Atlantic salmon populations to near zero (Hjeltnes 2014; Norris 2017). As such, the use of QTL in breeding programs has highlighted the power of genetic approaches to tackle infectious diseases by improving the disease resistance of aquaculture species. While the size of the effect of this QTL is not as large as for the IPN case, our results highlight a promising avenue to reduce the TiLV-related mortalities through the implementation of selection based on genetic markers in the QTL region. MAS for TiLV resistance could be performed without additional data recording, or in combination with TiLV challenge data on relatives of broodstock. It has been shown that the use of genetic markers together with family selection can achieve higher genetic gain (Spelman and Bovenhuis 1998) compared with the classic pedigree-based approach alone. In addition, future studies to elucidate the underlying causative gene and mutation would provide a putative target for CRISPR-Cas9 genome editing, which has significant potential to expedite the genetic improvement of disease resistance in aquaculture species (Gratacap et al. 2019).

## Supplementary information

Supplementary Figures

Supplementary Table 1

## Data Availability

Details of the SNPs used in the genome-wide association study are available in Peñaloza et al. (2020). The phenotype and genotype data supporting this study belongs to a breeding program managed and owned by WorldFish and is available upon reasonable request. Table S1 contains the complete list of candidate genes flanking the significant SNPs not shown on the main text. Figure S1 shows the Manhattan plot for TD, whereas the QQ plot for BS and TD is shown in Fig. S2.
