# Correction to: A major quantitative trait locus affecting resistance to Tilapia lake virus in farmed Nile tilapia (*Oreochromis niloticus*)

**DOI:** 10.1038/s41437-021-00461-6

**Published:** 2021-08-18

**Authors:** Agustin Barría, Trọng Quốc Trịnh, Mahirah Mahmuddin, Carolina Peñaloza, Athina Papadopoulou, Ophelie Gervais, V. Mohan Chadag, John A. H. Benzie, Ross D. Houston

**Affiliations:** 1 The Roslin Institute and Royal (Dick) School of Veterinary Studies, University of Edinburgh Easter Bush, Midlothian, UK; 2 WorldFish, Bayan Lepas, Malaysia

**Keywords:** Quantitative trait loci, Quantitative trait, Genetic markers, Animal breeding, Genome-wide association studies

Correction to: *Heredity*; 10.1038/s41437-021-00447-4

Due to a processing error, the presentation of Figure 1 was incomplete in pdf.

The original article has been corrected.

