# Supplementary material for: A major quantitative trait locus affecting resistance to Tilapia lake virus in farmed Nile tilapia (Oreochromis niloticus)
Source: Heredity (Edinb). 2021 Jul 14;127(3):334–43. doi: 10.1038/s41437-021-00447-4 (PMC8405827; doi:10.1038/s41437-021-00447-4)
Supplement: Supplementary file 1 — Supplementary Figures [file 41437_2021_447_MOESM1_ESM.pdf]

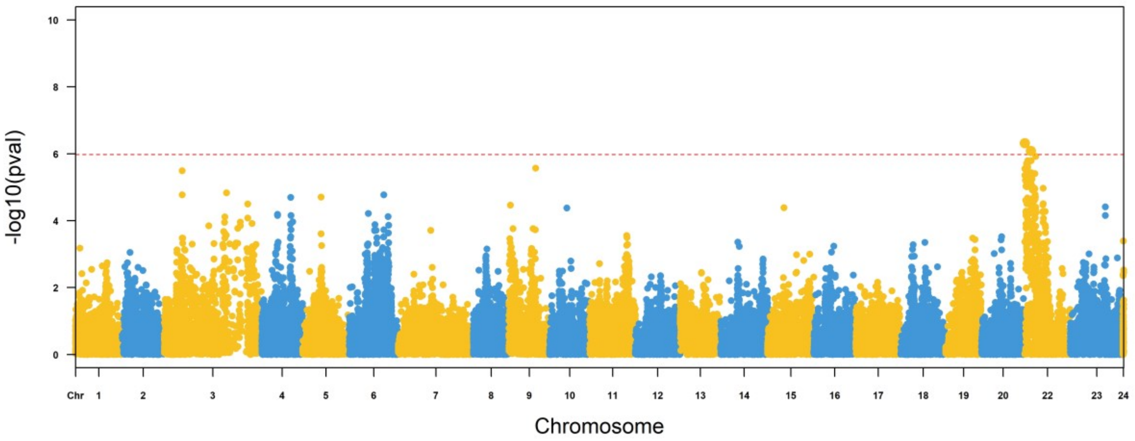

Figure S1. Manhattan plot for resistance to Tilapia Lake Virus (TiLV) in a Nile tilapia (*Oreochromis niloticus*) breeding population. Manhattan plot of GWAS for host resistance, as time to death, to TiLV. On the y axis is the –log10(P-value). Horizontal dashed red line shows the genome-wide significance threshold. Oni24 represent SNPs with unknown chromosome location

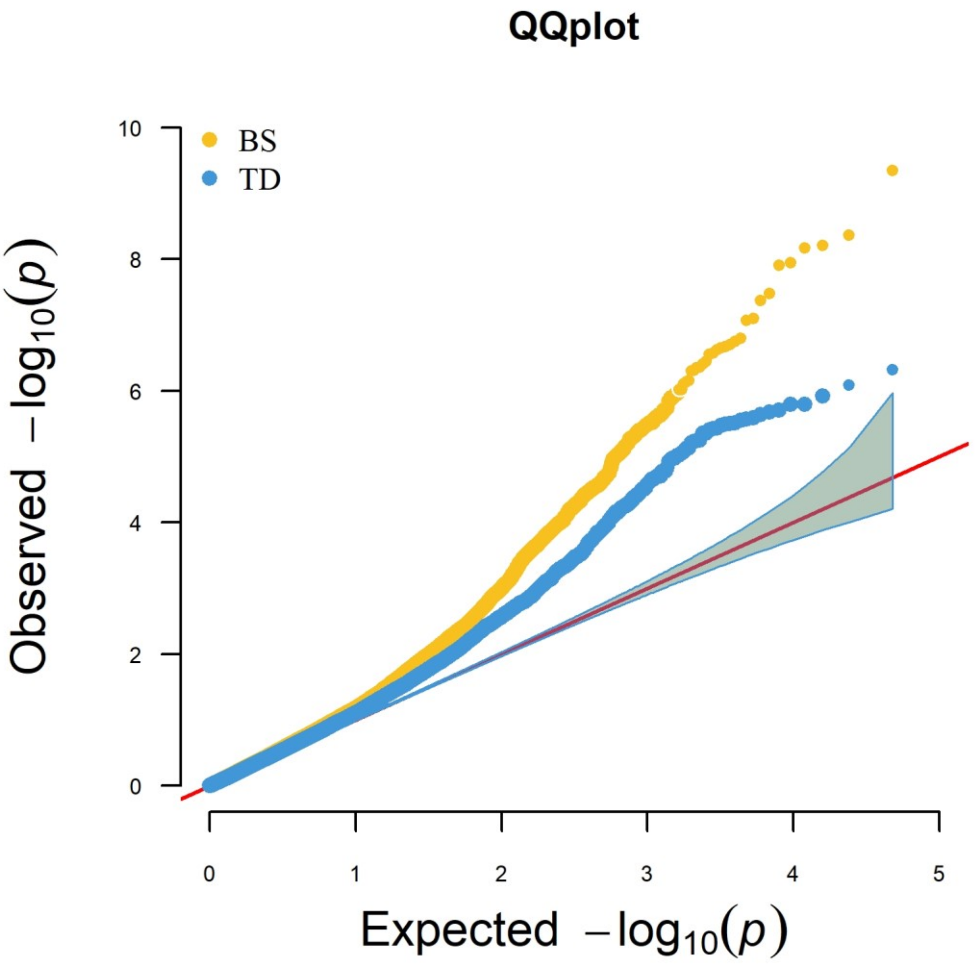

Figure S2. Quantile-quantile plot of genome-wide association for resistance to Tilapia Lake Virus (TiLV) in a Nile tilapia (*Oreochromis niloticus*) breeding population. The observed vs expected P-values are shown for host resistance to TiLV as binary survival (yellow) and time to death (blue).
